# Establishment of an Indirect ELISA Method for Detecting Multiple Virulence Factors from Porcine Diarrhea-Related *Escherichia coli* Based on a Multi-Epitope Fusion Antigen

**DOI:** 10.3390/vetsci13070684

**Published:** 2026-07-14

**Authors:** Shiyu Zhang, Sheng Lu, Jianan Liu, Zhonghao Chen, Caiying Li, Jiale Ma, Min Sun, Xinming Pan

**Affiliations:** 1 MOE Joint International Research Laboratory of Animal Health and Food Safety, College of Veterinary Medicine, Nanjing Agricultural University, Nanjing 210095, China; zhangshiyu@stu.njau.edu.cn (S.Z.); luzhi@stu.njau.edu.cn (S.L.); 2023807173@stu.njau.edu.cn (Z.C.); 2025207035@stu.njau.edu.cn (C.L.); jialema@njau.edu.cn (J.M.); 2 Key Lab of Animal Bacteriology, Ministry of Agriculture, Nanjing 210095, China; 3 Bacterial Pathogenesis Research Group, WOAH Reference Lab for Swine Streptococcosis, Nanjing 210095, China; 4 Institute of Veterinary Medicine, Jiangsu Academy of Agricultural Sciences, Nanjing 210000, China

**Keywords:** porcine diarrheagenic *E. coli*, multiepitope fusion antigen, virulence factors, indirect ELISA, serological surveillance

## Abstract

Porcine diarrheagenic *Escherichia coli* strains exhibit heterogeneous virulence profiles involving multiple factors, complicating broad-spectrum serological diagnosis. Here, we designed a multiepitope fusion antigen, MEAET, containing predicted B-cell epitopes from six major virulence factors, K88/F4, F18, LT, Stx2e, HlyA, and Tir, and developed an indirect ELISA for broad antibody detection. Structural modeling predicted a well-folded conformation with multiple discontinuous B-cell epitopes, and molecular docking suggested favorable interactions between the C-terminal dendritic cell-targeting peptide and the porcine immune receptor SLA1. Immunization of piglets with purified recombinant MEAET generated hyperimmune serum with a titer exceeding 1:102,400, which specifically recognized all six individual antigens by Western blot. The optimized ELISA demonstrated satisfactory repeatability (intra-assay CV ≤ 4.26%) and reproducibility (inter-assay CV ≤ 5.57%), showed no detectable cross-reactivity with antibodies against other tested porcine pathogens, and exhibited good analytical sensitivity, with positive sera detectable at dilutions up to 1:3200. The optimized ELISA showed good repeatability and reproducibility, with intra-assay and inter-assay coefficients of variation of ≤4.26% and ≤5.57%, respectively, no detectable cross-reactivity with the tested heterologous controls, and good analytical sensitivity, with positive sera remaining detectable at dilutions up to 1:3200. The S/P ratio cut-off was 0.203. In field sera, positivity rates were 74.5% (35/47) in diarrheic pigs and 34.0% (36/106) in asymptomatic pigs. These primary results indicate that the MEAET-based indirect ELISA represents a promising tool for serological detection of antibodies against multiple virulence factors of diarrheagenic *E. coli* in pigs and for field surveillance in swine herds.

## Simple Summary

Diarrheagenic *Escherichia coli* strains in pigs harbor diverse toxins and adhesins, which complicates broad-spectrum serological detection. To address this issue, we designed a recombinant multiepitope antigen containing epitopes derived from six major virulence factors and developed an indirect ELISA for antibody detection in porcine sera. Immunization of piglets with the recombinant antigen induced a strong humoral response, and the resulting antisera recognized all six corresponding individual antigens. The assay showed good repeatability and specificity, with no detectable cross-reactivity with antibodies against other common porcine pathogens. Application of the assay to field serum samples showed a higher positivity rate in diarrheic pigs than in asymptomatic animals, indicating that the method can effectively identify prior exposure to pathogenic *E. coli* strains carrying these virulence determinants. Overall, this MEAET-based indirect ELISA provides a promising tool for broad-spectrum serological surveillance of diarrheagenic *E. coli* infections in swine herds and may facilitate disease monitoring and control.

## 1. Introduction

*Escherichia coli* encompasses a diverse group of pathotypes [1], among which enteropathogenic *E. coli* (EPEC), enterohemorrhagic *E. coli* (EHEC), enterotoxigenic *E. coli* (ETEC), enteroaggregative *E. coli* (EAEC), and enteroinvasive *E. coli* (EIEC) represent the five major pathotypes responsible for intestinal disease [2]. ETEC is a leading cause of bacterial diarrhea in both humans and young livestock. In neonatal piglets, ETEC infection commonly presents as yellow or white scours and is associated with high morbidity and mortality [3,4].

Diarrheagenic *E. coli* strains responsible for piglet diarrhea typically exert their pathogenic effects through the coordinated action of multiple virulence factors. These include enterotoxins, such as STa, STb, and LT [5]; hemolysin encoded by the HlyCABD operon; Shiga toxins Stx1 and Stx2 [6]; and fimbrial adhesins, including F4 (K88) and F18 [7]. In addition, the translocated intimin receptor (Tir) and its bacterial ligand intimin are well-established contributors to pathogenicity [8]. In enterotoxigenic strains, fimbria-mediated adherence to the porcine small intestinal epithelium is followed by toxin release, which stimulates excessive fluid secretion into the intestinal lumen, leading to severe watery diarrhea and subsequent dehydration.

Recent advances in genomic sequencing and analysis have facilitated the identification of an increasing number of pathogenic *E. coli* subtypes and hybrid pathotypes in swine-derived samples [9]. This growing genetic and pathogenic diversity has complicated the epidemiology, pathogenesis, and control of swine-diarrhea-associated *E. coli* [10], highlighting the need for efficient, broad-spectrum detection methods. Although molecular methods such as PCR and whole-genome sequencing (WGS) offer high sensitivity and resolution for genotypic characterization, they require specialized equipment, are relatively costly, and are less practical for large-scale herd-level monitoring [11]. In contrast, serological assays, particularly ELISA, are rapid, cost-effective, and suitable for high-throughput screening, making them valuable complementary tools for field surveillance and exposure assessment. However, the extensive repertoire of toxins and fimbrial adhesins carried by *E. coli*, together with the diverse combinations of their encoding genes, makes it difficult to detect most virulence factors simultaneously using a single routine assay. This limitation poses a significant challenge to the accurate assessment of health status in swine herds. Although enzyme-linked immunosorbent assay (ELISA) is rapid and suitable for large-scale screening, making it a valuable tool for serological monitoring [12], its application to swine-diarrhea-associated *E. coli* is hindered by the difficulty of obtaining a single fusion antigen capable of detecting antibodies against multiple virulence factors. Specifically, STa and STb were excluded from the antigen design in this study due to their poor immunogenicity as small-molecule haptens, which complicates the generation of high-affinity antibodies. Moreover, the structural homology of STa to the endogenous mammalian hormones guanylin and uroguanylin raises potential autoimmunity concerns [13].

To address this challenge, the present study aimed to construct a multiepitope fusion antigen incorporating the major toxins and fimbrial adhesins of swine-diarrhea-associated *E. coli*. By optimizing the design and arrangement of antigenic epitopes, we developed a fusion antigen suitable for establishing a one-step ELISA-based detection system. This system could be further developed into a diagnostic kit, thereby improving detection efficiency and facilitating broader application in both laboratory and field settings.

## 2. Materials and Methods

### 2.1. Ethical Approval

The immunization of SPF piglets was performed at the Experimental Animal Center of Nanjing Agricultural University, in compliance with the animal welfare guidelines of the Animal Research Committee of Jiangsu Province, and was approved by the institutional ethics committee (Approval: NJAU.No20240528102, Date: 28 May 2024).

### 2.2. Collection of Clinical Serum Samples

The clinical sera samples used in this study, including 16 positive sera, 47 sera from diarrheal pigs, and 106 sera from asymptomatic pigs, were collected from 30-day-old weaned piglets from 17 pig farms in Jiangsu, Anhui, Jiangxi, and Zhejiang provinces, respectively. None of the pigs had been vaccinated against *Escherichia coli*. The negative serum panel consisted of 24 serum samples collected from healthy pigs with no clinical signs of diarrhea. These sera showed no detectable reactivity against the MEAET antigen or the selected virulence factor antigens by Western blot at a dilution ratio of 1:100 and were therefore defined as confirmed negative sera.

### 2.3. Gene Synthesis and Recombinant Plasmid Construction

A codon-optimized nucleotide sequence encoding MEAET (SEQ ID NO: 1 Table S1) was synthesized and cloned into a prokaryotic expression vector. The resulting recombinant plasmid was confirmed by sequencing and then transformed into *E. coli* BL21 (DE3). A positive clone was cultured in LB medium containing 100 μg/mL ampicillin until the OD600 reached 0.4–0.6, and protein expression was induced with 500 μM IPTG at 16 °C and 120 rpm for 18 h. The bacterial cells were harvested and lysed by sonication. The GST-tagged MEAET protein was purified by glutathione agarose affinity chromatography according to the manufacturer’s instructions, followed by thrombin digestion to remove the GST tag. The purified MEAET protein was analyzed by SDS-PAGE, and its concentration was determined using a BCA protein assay kit (Beyotime, Shanghai, China). The purified protein was stored at −80 °C until use.

### 2.4. Preparation of MEAET Protein-Specific Hyperimmune Serum

Purified MEAET protein was prepared as described above and emulsified with ISA 206 VG adjuvant at a volume ratio of 1:1 by thorough vortexing. Three 30-day-old SPF piglets were used for immunization by multiple-site intramuscular injection. The primary immunization dose was 1 mg per piglet, and the booster immunization dose was 2 mg per piglet. Booster immunizations were performed every 10 days, and a total of three immunizations were conducted. Blood samples were collected from the ear vein before immunization to obtain pre-immune serum as the negative control. After each immunization, blood samples were collected and sera were separated for antibody titer determination by ELISA. During the immunization period, piglets were monitored daily for general health status, appetite, behavior, body temperature, and local reactions at the injection sites. No piglets reached the predefined humane endpoints during the immunization experiment. When the antibody titer reached a high level (>1:102,400), blood was collected from the anterior vena cava, and the separated serum was used as positive serum for subsequent experiments. Serum samples were aliquoted into sterile EP tubes at 0.5 mL per tube, sealed, and stored at −40 °C until use.

### 2.5. Design and Construction of the Multiepitope Fusion Antigen

A multiepitope fusion antigen, designated MEAET, was designed to incorporate immunodominant B-cell epitopes derived from six key virulence factors associated with porcine diarrheagenic *E. coli*: hemolysin A (HlyA), Shiga toxin 2e (Stx2e), heat-labile enterotoxin (LT), the translocated intimin receptor (Tir), and the fimbrial subunits F18 and K88/F4. The candidate proteins were first predicted by linear epitope analysis through ABCpred server (https://webs.iiitd.edu.in/raghava/abcpred/ABC_submission.html, accessed on 12 July 2025) [14], and then the potential swine leukocyte antigen (SLA)-binding capacity was assessed by IEDB TepiTool (http://tools.iedb.org/tepitool/, accessed on 12 July 2025) [15] as an auxiliary criterion for immunogenicity evaluation. The final epitopes were ranked according to B-cell epitope score, antigenicity, hydrophilicity, surface accessibility, flexibility, GRAVY value, predicted SLA-binding potential, allergenicity/toxicity prediction, and sequence specificity. Based on this combined evaluation, two optimal epitopes from each target antigen were selected and linked using the flexible linker GPGPGLRMKLPKS, and a dendritic cell-targeting peptide, FYPSYHSTPQRP, was fused to the C-terminus to generate MEAET.

### 2.6. Development of an Indirect ELISA Using MEAET as the Coating Antigen

The purified MEAET antigen was evaluated as a coating antigen for an indirect ELISA for the simultaneous detection of antibodies against the six virulence factors. Optimal assay conditions were determined by checkerboard titration. Briefly, 96-well microtiter plates were coated with 100 μL per well of purified MEAET diluted in carbonate-bicarbonate buffer (pH 9.6). Coating carried out at 37 °C for 1 h followed by incubation at 4 °C overnight. After three washes with phosphate-buffered saline containing 0.05% Tween-20 (PBST), plates were blocked with 200 μL per well of 5% (*w*/*v*) skim milk in PBST at 37 °C for 2 h and then washed three times with PBST.

Porcine serum samples diluted 1:400 in PBST were added to the plates at 100 μL/well and incubated at 37 °C for 1 h. After three washes with PBST, horseradish peroxidase (HRP)-conjugated goat anti-pig IgG diluted 1:10,000 in PBST was added at 100 μL/well and incubated at 37 °C for 1 h. The plates were then washed five times with PBST, followed by the addition of 100 μL of 3,3′,5,5′-tetramethylbenzidine (TMB) substrate solution to each well. After incubation in the dark at 37 °C for 15 min, the reaction was stopped by adding 50 μL of 2 M H_2_SO_4_. The optical density (OD) was measured at 450 nm using a microplate reader.

### 2.7. ROC Analysis

Statistical calculations and graph plotting were performed using Python (version 3.10.1), and the ROC curve was generated using the matplotlib package (version 3.11.0). All analyses were conducted in the Python environment. The optimal cut-off threshold was determined based on the maximal Youden index (sensitivity + specificity − 1) and the receiver operating characteristic (ROC) curve. Experimental samples with OD values equal to or higher than the determined threshold value were considered serologically positive.

### 2.8. Analytical Sensitivity and Specificity Evaluation of the MEAET-Based ELISA

The sensitivity of the developed ELISA was evaluated by a two-fold serial dilution of three clinical positive serum samples screened in this study, The sera were diluted at 1:100, 1:200, 1:400, 1:800, 1:1600, 1:3200, 1:6400, and 1:12,800 with sample diluent and then tested by ELISA. The highest serum dilution at which the S/P value remained above the cut-off value of 0.203 was used to assess the analytical sensitivity of the assay. To evaluate the specificity of the method, ELISA was employed to analyze positive serum samples for three clinical positive serum samples screened in this study together with *Salmonella* spp. (Sal), *Pasteurella multocida* (PAS), *Clostridium perfringens* (CP), *Listeria monocytogenes* (LM), *Campylobacter jejuni* (CJ), *Streptococcus suis* (SS), non-pathogenic *Escherichia coli* K-12, porcine epidemic diarrhea virus (PEDV), and porcine deltacoronavirus (PDCoV). MEAET-positive serum and negative serum were used as positive and negative controls, respectively. All serum samples were diluted at 1:200, and each sample was tested in triplicate.

### 2.9. Determination of the Cut-Off Value and Assay Validation

To establish the cut-off value for seropositivity, a panel of confirmed negative serum samples was tested using the optimized indirect ELISA. The negative serum panel consisted of 24 serum samples collected from healthy pigs with no clinical signs of diarrhea, all of which showed no detectable reactivity against the MEAET antigen or the selected virulence factor antigens by Western blot. The cut-off value was calculated as the mean OD_450_ of the negative samples plus three standard deviations and was then converted to the corresponding sample-to-positive (S/P) ratio. Samples with an S/P ratio ≥ 0.203 were considered positive, whereas those with an S/P ratio < 0.203 were considered negative.

### 2.10. Statistical Analysis

All statistical analyses were performed using SPSS (version 31.0). Data are presented as mean ± standard deviation (SD). The cut off value was determined as described in Section 2.9. For comparison of seropositivity rates between diarrheic and asymptomatic pig groups, the chi square test was used as appropriate. A *p* value < 0.05 was considered statistically significant. The 95% confidence intervals for positivity rates were calculated using the Wilson score method. Repeatability and reproducibility CVs were calculated using standard formulas.

## 3. Results

### 3.1. Selection and Characterization of Predicted B Cell Epitopes

Porcine diarrheagenic *E. coli* strains typically colonize the intestinal epithelium via K88/F4 or F18 fimbriae and subsequently induce enteric pathology through multiple virulence factors, including heat-labile enterotoxin (LT), heat-stable enterotoxin (ST), hemolysin, Shiga toxin 2e (Stx2e), and type III secretion system effectors such as the translocated intimin receptor (Tir). Given the marked heterogeneity of virulence factor profiles among field isolates, strains associated with porcine diarrhea may encode various combinations of these determinants, complicating both pathogen detection and serological diagnosis. Because of its poor immunogenicity, ST was excluded from target antigen selection. Accordingly, six immunogenic proteins—HlyA, Stx2e, LT, Tir, F18, and K88/F4—were selected as target antigens.

B-cell epitopes were predicted from each antigen using BCPred and IEDB. After further screening based on antigenicity, amino acid composition, and physicochemical properties, including hydrophilicity, surface accessibility, and flexibility, two predicted optimal epitopes were retained from each protein (Table 1 and Table S2).

### 3.2. Design and In Silico Evaluation of the Multiepitope Fusion Antigen MEAET

**Figure 1:**
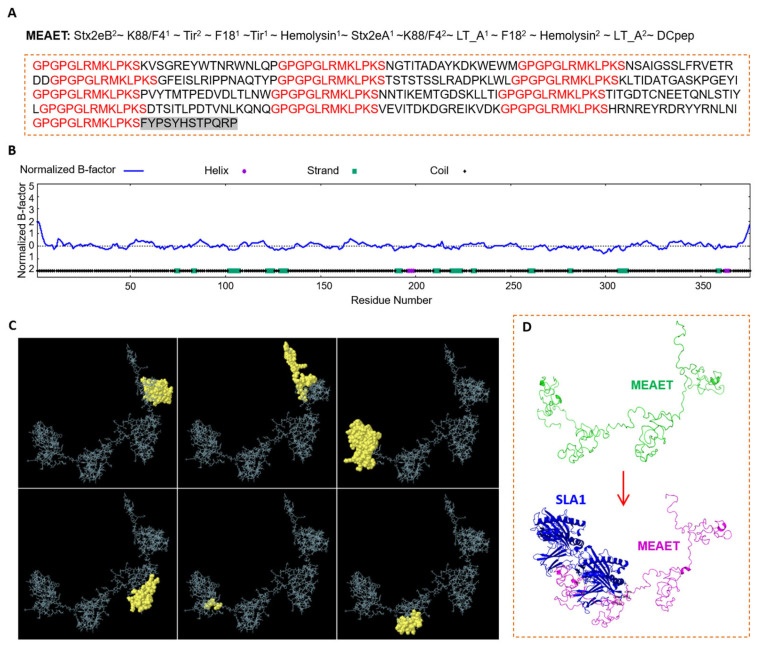
Design of the Multiepitope Fusion Antigen MEAET. (**A**) The MEAET multiepitope fusion antigen. Superscript numbers 1 and 2 indicate two different epitopes of the same protein, respectively. Red represents the linker peptide (as described in detail in the main text). (**B**) Secondary structure prediction. (**C**) DiscoTope analysis of conformational epitopes. Yellow indicates the spatial structure of individual epitopes. (**D**) Molecular docking simulation between MEAET and SLA1. The red arrow highlights the interaction result between MEAET and SLA1.

Hydrophilicity analysis of the candidate epitopes was performed using ExPASy ProtScale (https://web.expasy.org/protscale/, accessed on 12 July 2025). Based on the hydrophilicity indices, the selected epitopes were assembled in tandem using a flexible linker (GPGPGLRMKLPKS), and a dendritic cell-targeting peptide (FYPSYHSTPQRP) was fused to the C-terminus of the assembled epitope sequence. Multiple epitope arrangements were generated and comparatively evaluated for predicted immunogenicity, antigenicity, allergenicity, and physicochemical attributes. The arrangement with the most favorable overall profile was selected as the final construct (Figure 1A) and designated MEAET (Multiple-epitope antigen of *Escherichia coli* Virulence Factors). Its secondary structure was predicted (Figure 1B).

The three-dimensional structure of MEAET was predicted by homology modeling using AlphaFold 3 and further refined by energy minimization to obtain a thermodynamically stable conformation (Figure 1D). The quality of the final homology model was assessed by Ramachandran plot analysis (Figure S1). Discontinuous B-cell epitope analysis using DiscoTope revealed multiple conformational epitopes distributed across the folded structure (Figure 1C). To assess its potential for immune recognition, molecular docking simulations were performed between the refined MEAET model and the porcine immune recognition receptor SLA1. Molecular docking simulations suggested that the porcine immune receptor SLA1 may interact with the C-terminal dendritic cell-targeting peptide residues of the MEAET model mainly through its binding pocket, accompanied by predicted extensive hydrogen bond networks and hydrophobic interactions (Figure 1D).

### 3.3. Evaluation the Recognition Effect of MEAET Hyperimmune Serum for Different Antigens

**Figure 2:**
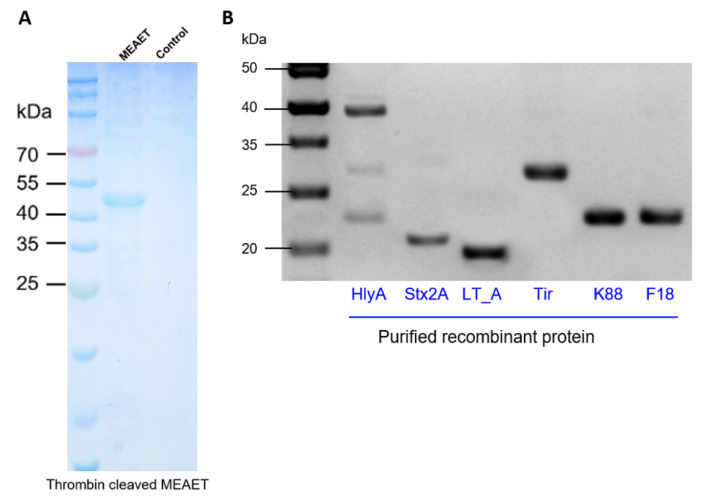
Preparation and Evaluation of the MEAET hyperimmune serum. (**A**) SDS-PAGE analysis of purified MEAET. The purified MEAET (~42 kDa), and empty vector control (Control) were resolved on a 12% SDS-PAGE gel with 10 μL sample loading per well and stained with Coomassie Blue. A dual-color prestained protein marker was used; the red band corresponds to 70 kDa and the green band to 25 kDa. (**B**) Western blot analysis of purified recombinant proteins using the MEAET hyperimmune serum. Each of the six recombinant proteins (HlyA, Stx2e, LT, Tir, K88/F4, and F18) was probed with the MEAET hyperimmune serum (1:100 dilution) and detected with HRP-conjugated anti-pig IgG. The blots were developed with enhanced chemiluminescence (ECL) substrate and exposed for 3 s. Molecular weight markers are indicated on the left.

The codon-optimized gene encoding MEAET (SEQ ID NO: 1, Table S1) was synthesized and cloned into the pGEX-4T-1 vector. The resulting recombinant plasmid was transformed into *E. coli* BL21 (DE3), and protein expression was induced with 500 μM IPTG at 16 °C for 18 h. The expressed GST-fused MEAET protein was purified by glutathione affinity chromatography, followed by removal of the GST tag through thrombin cleavage. SDS-PAGE analysis showed a single band corresponding to the expected molecular mass of approximately 42 kDa for the purified MEAET (Figure 2A and Table S2).

### 3.4. Generation and Characterization of MEAET Hyperimmune Serum

Purified MEAET was emulsified with ISA 206 VG adjuvant and administered to SPF piglets by intramuscular injection. After three immunizations, the antibody titer exceeded 1:102,400, as determined by ELISA. Hyperimmune serum was collected and stored at −40 °C for subsequent analyses. To evaluate the specificity of the MEAET-induced antibodies, the coding sequences of HlyA, Stx2e, LT, Tir, K88, and F18 were amplified by PCR, cloned into pET-28a(+), and expressed in *E. coli* BL21 (DE3). The purified recombinant proteins were subjected to Western blot analysis using MEAET hyperimmune serum. The serum specifically recognized all six antigens, yielding single bands at the expected molecular sizes (Figure 2B and Table S3).

### 3.5. Screening of Clinical Positive Sera

Using purified HlyA, Stx2e, LT, Tir, K88, and F18 proteins as antigens in Western blot assays, 16 clinical serum samples collected from convalescent piglets were identified as positive for antibodies against one or more of these virulence factors. The detailed reactivity profiles of these sera are summarized in Table 2. Among these samples, both single-antigen reactivity (e.g., samples 2, 3, and 10) and mixed antibody responses against multiple virulence factors (e.g., samples 4, 8, and 13) were observed, reflecting the heterogeneity of naturally acquired antibody responses under field conditions. These samples were stored at −40 °C for subsequent assay development.

An indirect ELISA using the MEAET antigen was successfully established. The optimal reaction conditions were determined as follows: coating antigen concentration, 1.0 μg/mL; serum dilution, 1:800; blocking solution, 5% skim milk; HRP-conjugated secondary antibody dilution, 1:10,000; and incubation time, 1 h for both primary and secondary antibodies (Tables S3–S6).

**Figure 3:**
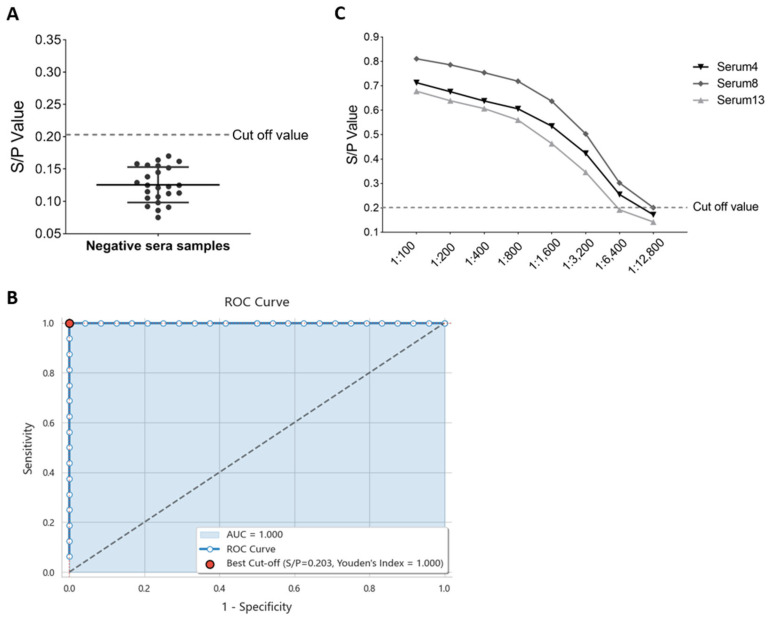
The analyses of cut-off value and sensitivity. (**A**) Establishment of the cut-off value based on negative serum samples. (**B**) Analytical sensitivity of the MEAET-based indirect ELISA. Serially diluted positive serum samples were tested under optimized conditions. The dashed line indicates the cut-off value. (**C**). ROC curve of the MEAET-based ELISA. AUC = 1; optimal cut-off = 0.203; sensitivity = 100%; specificity = 100%. The gray diagonal line indicates the baseline of random guessing.

To determine the cut-off value, 24 confirmed negative sera were tested. The S/P ratio cut-off was calculated as the mean S/P of negative samples plus 3 SD, yielding a threshold of 0.203. Samples with S/P ≥ 0.203 were considered positive (Figure 3A; Table S7), repeatability and reproducibility are shown in (Figure 3B; Table S8) and ROC analysis was performed to evaluate diagnostic performance (Figure 3C; Table S9).

### 3.6. Validation of the MEAET-Based Indirect ELISA

#### 3.6.1. Repeatability

Intra-assay and inter-assay reproducibility were evaluated using three serum samples. The intra-assay coefficients of variation (CV) ranged from 1.82% to 4.26%, while the inter-assay CVs ranged from 2.78% to 5.57% (Table 3). All CVs were below the 10% threshold, indicating satisfactory repeatability and reproducibility.

#### 3.6.2. Specificity

The optimized ELISA was used to test three clinical serum samples positive for the target antibodies (samples 4, 8, and 13), together with sera positive for other porcine pathogens, including *Salmonella* spp., *Pasteurella* spp., *Clostridium perfringens*, *Listeria monocytogenes*, *Campylobacter jejuni*, *Streptococcus* spp., non-pathogenic *E. coli* K12, PEDV, and PDCoV. All three clinical samples yielded S/P values above the cut-off, whereas all heterologous pathogen-positive sera produced S/P values below 0.203 (Table 4). These findings demonstrate that the MEAET-based ELISA specifically detects antibodies against the target *E. coli* virulence factors, with no detectable cross-reactivity with antibodies against the above other porcine pathogens.

#### 3.6.3. Sensitivity

Three clinical serum samples positive for the target antibodies (samples 4, 8, and 13) were serially diluted from 1:100 to 1:12,800 and tested using the optimized ELISA. All three samples remained positive at dilutions up to 1:3200 (Figure 3B), indicating good analytical sensitivity of the assay.

### 3.7. Application of the MEAET-Based ELISA to Clinical Samples

The established indirect ELISA was employed to screen 47 serum samples from diarrheic pigs, in which major viral infections had been excluded, and 106 samples from asymptomatic pigs. The seropositivity rate was 74.5% in diarrheic pigs (35/47; 95% CI: 60.5–84.7%) and 34.0% in asymptomatic pigs (36/106; 95% CI: 25.6–43.4%) (Table 5). The difference in seropositivity rates between the two groups was statistically significant (χ^2^ = 21.48, *p* < 0.001).

## 4. Discussion

In this study, we designed a multiepitope fusion antigen, MEAET, incorporating immunodominant B-cell epitopes from six major virulence factors associated with porcine diarrheagenic *E. coli*: HlyA, Stx2e, LT, Tir, F18, and K88/F4 [16]. The multiepitope fusion antigen strategy has been widely regarded as a rational approach for integrating multiple dominant antigenic targets into a single recombinant protein, making it particularly suitable for serological detection of pathogenic microorganisms with diverse virulence profiles [17]. The resulting recombinant protein was successfully employed as a coating antigen in an indirect ELISA, enabling the simultaneous detection of antibodies against multiple virulence determinants. This approach reduces the logistical complexity and cost associated with parallel testing using multiple single-antigen assays, thereby providing a practical and scalable approach for broad-spectrum serological surveillance of porcine diarrheagenic *E. coli* in swine herds [17,18]. In contrast to many multiepitope or MEFA-based constructs that are primarily developed as vaccine immunogens, the MEAET construct in this study was designed specifically as a serological screening antigen. Thus, the main rationale of MEAET was not to induce protective immunity, but to broaden antibody detection coverage using a single recombinant antigen in an indirect ELISA format.

The increasing genetic diversity and prevalence of hybrid pathotypes among swine-derived *E. coli* isolates have highlighted the limitations of conventional single-antigen ELISAs, which may fail to detect antibody responses induced by strains carrying non-target virulence factors [19]. The multiepitope fusion antigen (MEFA) platform addresses this diagnostic gap by assembling epitopes from multiple antigens into a single immunogen while preserving their individual antigenic integrity [20]. Compared with existing MEFA designs, the novelty of MEAET lies in its diagnostic-oriented antigen composition and structural design. First, the selected epitopes were derived from both toxin-associated antigens and colonization/adhesion-related antigens, allowing the assay to cover different virulence categories of porcine diarrheagenic *E. coli*. Second, the epitopes were selected through a combined in silico strategy considering predicted antigenicity, surface accessibility, and sequence specificity, rather than simply concatenating antigen fragments. Third, the use of a flexible linker was intended to minimize steric interference between adjacent epitopes and maintain epitope exposure within the fusion protein. In this study, in silico analyses indicated that the selected epitopes had favorable predicted antigenicity and surface accessibility, with minimal sequence homology to porcine proteins. This represents a critical quality control criterion for the rational design of epitope-based antigens in veterinary applications, as it may reduce the potential risk of autoimmune cross-reactivity [21]. Structural modeling further predicted a well-folded conformation containing multiple discontinuous B-cell epitopes. Molecular docking simulations revealed favorable interactions between the cytoplasmic tail dendritic cell-targeting peptide and the porcine immune receptor SLA1, suggesting a potential role for this peptide in facilitating antigen recognition and presentation [22]. Therefore, the MEAET design combines epitope selection, linker-mediated spatial separation, and immune-targeting peptide incorporation into a single diagnostic antigen framework. The selection of the six antigens incorporated in MEAET was based on their epidemiological relevance and immunodominance as major virulence determinants of porcine diarrheagenic *E. coli*, allowing broad yet practical coverage for routine serological surveillance while maintaining a construct size compatible with efficient recombinant expression.

Immunization of SPF piglets with purified MEAET elicited high-titer antibodies (>1:102,400) that specifically recognized all six parental virulence factors in Western blot assays, indicating that the assembled epitopes retained antigenic reactivity and did not exhibit obvious immune interference within the fusion antigen [23,24]. These findings are consistent with recent MEFA-based studies targeting porcine enteric bacterial and viral pathogens [24,25], and further extend this platform by simultaneously incorporating toxin- and fimbria-derived epitopes from porcine diarrheagenic *E. coli*. This distinguishes MEAET from single-target or single-pathotype antigen designs and supports its potential use as a broad-spectrum screening tool for detecting antibody responses associated with multiple virulence factors [26]. However, it should also be noted that the MEAET-based ELISA indicates exposure to one or more of the included virulence-associated antigens but cannot distinguish antibody responses against individual antigens. Therefore, MEAET should be regarded as a preliminary broad-spectrum serological screening antigen, and further diagnostic validation using independently confirmed clinical serum panels and established reference methods.

The optimized indirect ELISA demonstrated robust analytical performance. The optimal assay conditions were established as follows: coating antigen concentration of 1.0 μg/mL, serum dilution of 1:800, blocking with 5% skim milk, and incubation for 1 h with both primary and secondary antibodies [26]. The assay exhibited excellent repeatability and reproducibility, with intra- and inter-assay CVs below 6%, and showed high specificity, with no detectable cross-reactivity against the limited panel of other porcine pathogens tested. The assay also showed good analytical sensitivity, as positive sera remained detectable at dilutions up to 1:3200. Collectively, these performance characteristics are comparable to those required for preliminary assay validation in veterinary serology [27,28]; Although ROC analysis was performed to evaluate the diagnostic performance, the reference panels were limited in size and were not based on a definitive gold standard, which may affect the precision of the estimates of diagnostic sensitivity and specificity. Therefore, the diagnostic performance of the assay requires further validation using larger, well-characterized serum panels confirmed by reference-standard methods. Notably, the assay successfully detected antibodies in clinical sera from convalescent piglets with diverse reactivity profiles, including both single-antigen and multi-antigen antibody responses, such as responses against both LT and K88. These preliminary findings underscore its potential utility as a screening tool for field surveillance of porcine diarrheagenic *E. coli* infections with heterogeneous virulence profiles [17,26].

Application of the established ELISA to clinical samples revealed seropositivity rates of 74.5% in diarrheic pigs and 34.0% in asymptomatic pigs. This seroepidemiological pattern is consistent with field surveys of porcine diarrheagenic *E. coli* in major swine-producing regions [29]. The higher seropositivity rate in diarrheic animals appears to support an association of these virulence factors with the pathogenesis of porcine colibacillosis, as also indicated by genomic and functional studies of field isolates [30,31]. Meanwhile, the detection of antibodies in a substantial proportion of asymptomatic pigs suggests previous exposure to, or possible subclinical infection with, *E. coli* strains carrying these virulence factors. Such animals may contribute to the maintenance and transmission of pathogenic strains within and between swine herds, an epidemiological feature increasingly recognized as a challenge for the prevention and control of porcine colibacillosis [32]. The detection of multi-target antibody responses in some asymptomatic animals further indicates frequent exposure to diverse pathogenic strains, consistent with the extensive strain heterogeneity and frequent emergence of hybrid pathotypes of *Escherichia coli* reported in global swine populations [19]. Nevertheless, it should be interpreted with caution that seropositivity in asymptomatic pigs could also reflect detection of maternal antibodies, nonspecific background reactivity, or previous exposure without active infection, as the current dataset lacks bacteriological and molecular confirmation to distinguish these possibilities. Therefore, the observed positivity rates in both diarrheic and asymptomatic pigs should be interpreted as serological exposure signals rather than as definitive evidence that the targeted *E. coli* virulence factors caused the diarrhea.

Several limitations of this study should be considered. First, although in silico analyses indicated favorable predicted antigenic properties of the selected epitopes, the relative immunogenic contribution of each individual epitope within the MEAET construct requires further empirical validation in vivo. This represents a well-recognized limitation inherent to computational prediction-based rational design of multiepitope antigens [33,34]. Second, the exclusion of STa and STb inherently restricts the assay’s coverage of the complete virulence repertoire of porcine diarrheagenic *E. coli*, and thus the assay should not be claimed as comprehensive. In addition, the absence of paired bacteriological isolation and whole-genome characterization for the clinical serum samples precluded a definitive correlation between seropositivity and active infection status, as seroconversion may reflect either ongoing infection or previous exposure to pathogenic strains [27,35]. In addition, the repeatability and reproducibility evaluation was based on only three serum samples, which, while acceptable for preliminary assay validation, is not sufficient for full diagnostic validation and should be addressed in future studies with a larger sample set. Although ROC analysis was performed, the reference panels were limited and were not based on a definitive gold standard; therefore, the estimated diagnostic sensitivity and specificity should be interpreted with caution. Therefore, further validation using reference-standard confirmed clinical samples is required before its routine diagnostic application.

Future work should focus on three key directions to address these limitations and broaden the practical utility of this study. First, the diagnostic performance of the established ELISA should be validated using larger, well-characterized serum panels with paired bacteriological and clinical metadata, which is an essential prerequisite for assay standardization and commercial application in accordance with WOAH guidelines [27,36]. Second, the dynamic changes of antibody levels induced by MEAET and individual virulence factors should be assessed following experimental infection with well-defined *E. coli* strains. Such studies would help clarify the optimal detection window of the assay and the quantitative relationship between antibody titers and infection status [37]. Finally, the immunoprotective efficacy and vaccine potential of MEAET should be evaluated in piglet challenge studies to explore its dual value as both a broad-spectrum diagnostic antigen and a novel subunit vaccine candidate against porcine diarrheagenic *E. coli* [16].

## 5. Conclusions

In summary, we designed and characterized a multiepitope fusion antigen encompassing immunodominant B-cell epitopes from six major virulence factors of porcine diarrheagenic *E. coli*. MEAET exhibited favorable structural properties, elicited broadly reactive antibodies in immunized piglets, and served as an effective coating antigen for an indirect ELISA capable of simultaneously detecting antibodies against multiple virulence determinants. Application of this assay to clinical samples revealed substantial seroprevalence in both diarrheic and asymptomatic pigs, highlighting its utility for comprehensive seroepidemiological surveillance. This MEFA-based strategy offers a versatile platform for developing broad-spectrum diagnostic tools and, potentially, multivalent vaccines against antigenically heterogeneous pathogens.

## Figures and Tables

**Table 1:** Information of candidate epitopes.

Antigen	Sequence	Position (aa)	B Score	Binding Alleles	Antigen Score	Gravy
Hemolysin ^1^	KLTIDATGASKPGEYI	635–650	0.93	10	0.9644	−0.269
Hemolysin ^2^	VEVITDKDGREIKVDK	911–926	0.9	6	1.6213	−0.825
Stx2eA ^1^	PVYTMTPEDVDLTLNW	209–224	0.92	11	1.1776	−0.225
Stx2eB ^2^	KVSGREYWTNRWNLQP	41–56	0.96	9	1.5262	−1.594
LT_A ^1^	TITGDTCNEETQNLSTIYL	211–229	0.96	24	1.0399	−0.416
LT_A ^2^	HRNREYRDRYYRNLNI	158–173	0.91	9	1.0155	−2.425
Tir ^1^	TSTSTSSLRADPKLWL	210–225	0.88	13	1.2372	−0.406
Tir ^2^	NSAIGSSLFRVETRDD	101–116	0.87	9	0.7514	−0.588
F18 ^1^	GFEISLRIPPNAQTYP	140–155	0.95	21	1.3503	−0.350
F18 ^2^	DTSITLPDTVNLKQNQ	190–205	0.94	21	0.5716	−0.819
K88/F4 ^1^	NGTITADAYKDKWEWM	34–49	0.82	16	0.8934	−1.044
K88/F4 ^2^	NNTIKEMTGDSKLLTI	57–72	0.83	17	0.5807	−0.412

Note: “1”; “2” Superscript numbers 1 and 2 indicate two different epitopes of the same protein, respectively.

**Table 2:** Characteristics of the 16 clinical serum samples used in this study.

Samples	Anti-HlyA	Anti-Stx2A	Anti-LT_A	Anti-Tir	Anti-K88	Anti-F18
1	−	−	−	−	+	−
2	+	−	−	−	−	−
3	−	+	−	−	−	−
4	−	−	+	−	+	−
5	−	−	+	−	−	−
6	−	−	−	−	+	−
7	−	−	+	−	−	−
8	−	+	+	−	+	−
9	+	−	−	−	−	−
10	−	−	−	−	−	+
11	+	+	−	−	−	−
12	−	−	+	−	+	−
13	−	−	+	−	−	+
14	−	−	+	−	+	−
15	−	−	+	−	−	+
16	−	−	−	+	−	−

Note: “+” represent the present; “−” represent the absent.

**Table 3:** Intra-assay and inter-assay reproducibility.

Serum Samples	Intra-Assay (*n* = 3)		Serum Samples	Inter-Assay (*n* = 3)	
	X ± SD	CV%		X ± SD	CV%
1	0.557 ± 0.024	4.26%	4	1.471 ± 0.059	5.57%
2	0.366 ± 0.018	1.82%	5	1.288 ± 0.041	3.85%
3	1.271 ± 0.045	3.39%	6	1.252 ± 0.032	2.78%

**Table 4:** Specificity of the MEAET-based indirect ELISA.

Samples	OD_450_	S/P	Interpretation	Samples	OD_450_	S/P	Interpretation
αSal	0.341	0.121	Negative	αPEDV	0.243	0.063	Negative
αPAS	0.319	0.108	Negative	αPDCoV	0.230	0.055	Negative
αCP	0.306	0.100	Negative	Serum sample 4	1.162	0.606	Positive
αLM	0.294	0.093	Negative	Serum sample 8	1.352	0.719	Positive
αCJ	0.282	0.086	Negative	Serum sample 13	1.084	0.560	Positive
αSS	0.268	0.078	Negative	Positive serum	2.061	/	/
αK12	0.336	0.118	Negative	Negative serum	0.137	/	/

**Table 5:** Clinical sample analysis by MEAET-based indirect ELISA.

Sample Type	Positive	Negative
Diarrheic pigs (*n* = 47)	74.5% (35/47; 95% CI: 60.5–84.7%)	25.5% (12/47)
Asymptomatic pigs (*n* = 106)	34% (36/106; 95% CI: 25.6–43.4%)	66% (70/106)

## Data Availability

The original contributions presented in this study are included in the article/Supplementary Material. Further inquiries can be directed to the corresponding authors.

## Supplementary Materials

The following supporting information can be downloaded at: https://www.mdpi.com/article/10.3390/vetsci13070684/s1, Table S1. Codon-optimized nucleotide sequence of the MEAET antigen; Table S2. Predicted properties of screened B cell epitopes; Table S3. Checkerboard titration for determining optimal assay conditions; Table S4. Optimization of blocking conditions for MEAET-based indirect ELISA; Table S5. Optimization of secondary antibody dilution; Table S6. Determination of optimal incubation times for primary and secondary antibodies; Table S7. OD_450_ values of the 24 negative serum samples; Table S8. Raw data for repeatability and reproducibility evaluation; Table S9. Raw data for the ROC curve analysis; Figure S1. Ramachandran plot of the refined MEAET homology model; Figure S2. Uncropped SDS-PAGE gel of the purified recombinant proteins; Figure S3. Uncropped Western blot images of the six recombinant proteins.
