# Supplementary material for: Establishment of an Indirect ELISA Method for Detecting Multiple Virulence Factors from Porcine Diarrhea-Related Escherichia coli Based on a Multi-Epitope Fusion Antigen
Source: Vet Sci. 2026 Jul 14;13(7):684. doi: 10.3390/vetsci13070684 (PMC13418581; doi:10.3390/vetsci13070684)
Supplement: Supplementary file 1 [file vetsci-13-00684-s001.zip › vetsci-4357740-supplementary.pdf]

Table S1. Codon-optimized nucleotide sequence of the MEAET antigen.

Sequence name	Nucleotide sequence (5'→3')
SEQ ID NO.1	GGGCCAGGTCCCGGATTAAGGATGAAACTACCGAAATCTCACCGT<br>AACCGTGAGTACCGTGATCGTTATTACCGCAACCTGAATATTGGTC<br>CAGGGCCGGGTCTGCGCATGAAGTTGCCAAAATCCAACGGTACCA<br>TTACCGCAGACGCGTATAAAGACAAGTGGGAATGGATGGGTCCGG<br>GTCCGGGCTTGCGCATGAAGCTGCCTAAGTCTGATACCTCCATTAC<br>TCTGCCAGACACGGTTAATCTGAAACAAAACCAGGGTCCGGGTCC<br>GGGCCTCCGTATGAAATTGCCAAAGAGCGGCTTTGAAATCAGCCTG<br>CGTATTCCGCCAAATGCGCAGACCTATCCGGGACCGGGTCCTGGCT<br>TACGCATGAAACTGCCGAAATCAAACAACACCATCAAAGAGATGA<br>CCGGCGACAGCAAGTTGCTGACCATCGGTCCCGGCCCGGGTCTGCG<br>AATGAAATTGCCGAAGTCCAAGTTGACGATCGACGCGACCGGTGC<br>TAGCAAGCCGGGCGAATACATCGGCCCGGGCCCGGGTTTACGCAT<br>GAAATTGCCGAAGTCGCCGGTTTATACCATGACTCCGGAAGATGTA<br>GACCTGACCCTGAATTGGGGTCCAGGCCCGGGCTTGCGTATGAAGC<br>TTCCGAAATCCACCAGCACGAGCACCTCTAGCCTGCGCGCGGATCC<br>GAAACTCTGGCTGGGTCCGGGTCCAGGCCTGAGAATGAAGCTGCC<br>GAAATCTACGATCACCGGTGACACGTGCAACGAAGAAACCCAAAA<br>CCTGAGCACGATCTACCTGGGTCCGGGTCCGGGCCTCCGGATGAAA<br>CTCCCGAAGTCCAACAGCGCCATTGGCAGCAGCCTGTTCCGTGTGG<br>AGACACGTGATGATGGCCCGGGCCCGGGGCTCCGTATGAAACTGC<br>CGAAGTCCGTGGAGGTTATTACCGACAAGGATGGTCGTGAGATCA<br>AAGTCGACAAAGGTCCGGGCCCGGGCCTGCGTATGAAGCTGCCGA<br>AAAGCAAGGTGAGCGGTCGCGAGTACTGGACCAATCGTTGGAATC<br>TGCAGCCGGGCCCGGGCCCGGGGCTTCGTATGAAGCTGCCGAAGT<br>CGTTCTATCCGAGTTACCATAGCACTCCGCAACGTCCG

Table S2. Predicted properties of screened B-cell epitopes.

Rank	Sequence	Start position	End position	B Score	No. of binding alleles	Antigen score	
**HlyA (Hemolysin)**							
1	KLTIDATGASKPGEYI	635	650	0.93	10	0.9644	√
1	ASLIGAPISMLVSALT	374	389	0.93	19	-0.0194	
2	KETGAIDAALTTINTVLS	345	362	0.92	14	0.4292	
3	GGEGNDYLNGGFGNDI	816	831	0.91	5	1.1226	
3	GGTGDDKLYGGGGIDL	798	813	0.91	1	1.8877	
3	SGGQGDDQLFGGSGND	752	767	0.91	1	1.6872	
4	VEVITDKDGREIKVDK	911	926	0.9	6	1.6213	√
4	GDDGDDYISGGQGDDQ	744	759	0.9	3	1.3823	
4	FEFRTGGIPYDVIDNLHSV	685	700	0.9	11	0.3682	
**Stx2e A**							
1	PECQITGDRPVIKINN	280	295	0.94	9	1.2959	
2	HISQGATSVSVINHTPPGSYISV	52	74	0.8	25	0.3934	
2	PVYTMTPEDVDLTLNW	209	224	0.92	11	1.1776	√
**Stx2e B**							
1	KVSGREYWTNRWNLQP	41	56	0.96	9	1.5262	√
2	ADCAKGKIEFSKYNED	20	35	0.82	12	0.6931	
**LT_A**							
1	TITGDTCNEETQNLSTIYL	211	229	0.96	24	1.0399	√
2	RGTQTGFVRYDDGYVSTSL	64	82	0.93	20	0.4318	
3	HRNREYRDRYYRNLNI	158	173	0.91	9	1.0155	√
**Tir**							
1	HFSGNSPVTGRLVGTP	476	491	0.95	4	0.7857	
2	TIAAGLIGMAATGIAQAVAL	229	248	0.94	10	0.3012	
3	STGTVENPYADVGMPRNDSL	434	453	0.92	15	-0.045	
3	TPEPDDPITTDPDAAA	249	264	0.92	3	0.8972	
4	SEEPIYDEVAADPNYSV	457	473	0.91	9	0.3547	
4	EQTITTRTVVDNQPTN	383	398	0.91	4	0.7085	
5	PGLPTNPLRFAASEVS	68	83	0.9	10	0.3894	
5	NQLINSNGPMGSRLLF	34	49	0.9	5	0.2397	
5	GGRGGAGHAMVTVASD	157	172	0.9	1	1.9412	
7	TSTSTSSLRADPKLWL	210	225	0.88	13	1.2372	√
7	GGGIGAGVTAALHRKN	364	379	0.88	6	1.4032	
7	TSGPEESPASRRNSNASL	408	425	0.88	6	1.2519	
8	NSAIGSSLFRVETRDD	101	116	0.87	9	0.7514	√
**F18**							
1	GFEISLRIPPNAQTYP	140	155	0.95	21	1.3503	√
2	DTSITLPDTVNLKQNQ	190	205	0.94	21	0.5716	√
3	APSVSNTITGIPNQND	215	230	0.91	8	0.0272	
4	KRSITMKVVKSSMINF	265	280	0.88	14	0.536	
**K88-F4**							
1	EWMVGGALSFNNTIKE	47	62	0.92	11	0.1952	
1	GQTIEATFTNPVVSTT	250	265	0.92	2	0.6222	

2	SYSEISTGLVGITSVAS	152	167	0.91	15	0.2213	
3	AVNPNAGNRGQVNKNS	210	225	0.88	2	1.4436	
3	AAHAWTTGDFNGSFDM	18	33	0.88	12	0.598	
4	VIASSYALGIDQGQTI	238	253	0.84	4	0.4075	
4	FFELPMKDDSGNNLGS	124	139	0.84	4	0.94	
5	NNTIKEMTGDSKLLTI	57	72	0.83	17	0.5807	√
6	NGTITADAYKDKWEWM	34	49	0.82	16	0.8934	√
7	TSVASGDNTSIYYGGL	163	178	0.81	21	0.5051	
7	PLIAFSDYEGNGVALQ	100	115	0.81	5	1.0469	

Table S3. Checkerboard titration for determining optimal assay conditions.

Coating concentration μg/mL		Serum dilution					
		1:400	1:800	1:1600	1:3200	1:6400	1:12800
2	P	2.165	2.133	1.408	1.058	0.713	0.445
	N	0.236	0.161	0.124	0.104	0.098	0.094
	P/N	9.173	13.248	11.354	10.173	7.275	4.734
1	P	2.155	2.027	1.401	1.033	0.682	0.356
	N	0.203	0.137	0.113	0.093	0.091	0.086
	P/N	10.615	**14.795**	12.398	11.107	7.494	4.139
0.5	P	1.866	1.816	1.213	0.702	0.553	0.314
	N	0.218	0.148	0.116	0.095	0.089	0.087
	P/N	8.679	12.270	10.457	7.389	6.213	3.609
0.25	P	1.167	1.021	0.603	0.351	0.229	0.151
	N	0.193	0.141	0.107	0.086	0.085	0.083
	P/N	6.046	7.241	5.635	4.081	2.694	1.819

Table S4. Optimization of blocking conditions for MEAET-based indirect ELISA.

	1% BSA	2% BSA	5% skim milk	10% skim milk
P	1.757	2.011	2.115	1.703
N	0.178	0.219	0.142	0.174
P/N	9.871	9.182	**14.894***	9.787

Table S5. Optimization of secondary antibody dilution.

	1:5000	1:10000	1:15000	1:20000
P value	2.015	2.126	1.805	1.557
N value	0.183	0.146	0.142	0.136

P/N	11.011	**14.562***	12.711	11.448

Table S6. Determination of optimal incubation times for primary and secondary antibodies.

Secondary Ab incubation (h)		Primary Ab incubation (h) 0.5	1.0	1.5	2.0
	P	1.838	1.935	1.974	2.088
0.5	N	0.134	0.145	0.151	0.159
	P/N	13.716	13.344	13.072	13.132
	P	1.928	2.108	2.032	2.109
1.0	N	0.139	0.143	0.151	0.159
	P/N	13.870	**14.741***	13.456	13.264
	P	1.939	2.113	2.088	2.156
2.0	N	0.151	0.163	0.170	0.179
	P/N	12.841	12.963	12.282	12.044

Table S7. OD_{450} values of the 24 negative serum samples.

Sample ID	Negative sera samples
N1	0.086
N2	0.125
N3	0.098
N4	0.112
N5	0.156
N6	0.138
N7	0.145
N8	0.113
N9	0.164
N10	0.105
N11	0.092
N12	0.155
N13	0.091
N14	0.107
N15	0.115
N16	0.121
N17	0.152
N18	0.158
N19	0.162
N20	0.075
N21	0.123

N22	0.129
N23	0.17
N24	0.125

[1] The negative serum panel (n = 24) was obtained from healthy 30-day-old weaned piglets from farms in Jiangsu, Anhui, Jiangxi, and Zhejiang provinces. All animals had no clinical signs of diarrhea or history of *E. coli* vaccination. All sera showed no detectable reactivity against MEAET or individual virulence factor antigens by Western blot at a 1:100 dilution, and were thus defined as confirmed negative.

Table S8. Raw data for repeatability and reproducibility evaluation.

	Serum 4	Serum 8	Serum 13
1:100	0.713	0.811	0.678
1:200	0.676	0.786	0.639
1:400	0.638	0.754	0.607
1:800	0.606	0.719	0.56
1:1600	0.535	0.637	0.463
1:3200	0.423	0.503	0.346
1:6400	0.255	0.302	0.192
1:12800	0.172	0.201	0.142

Table S9. Raw data for the ROC curve analysis.

negative	positive
0.086	0.156
0.125	0.289
0.098	0.277
0.112	0.606
0.156	0.254
0.138	0.208
0.145	0.303
0.113	0.719
0.164	0.384
0.105	0.366
0.092	0.437
0.155	0.416
0.091	0.56
0.107	0.433
0.115	0.394
0.121	0.169
0.152	
0.158	
0.162	
0.075	
0.123	
0.129	
0.17	
0.125	

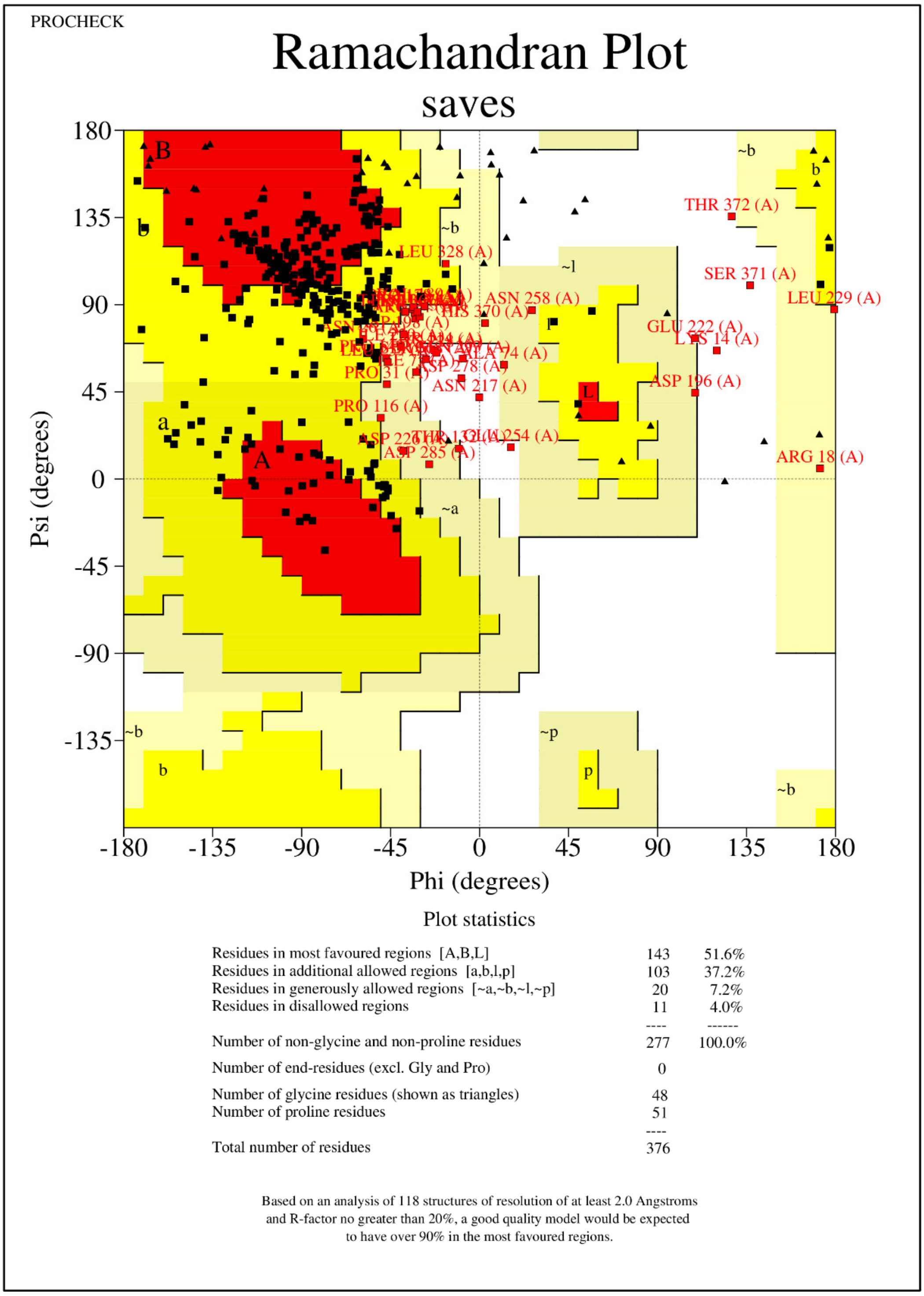

saves_01.ps

Supplementary Figure S1. Ramachandran plot of the refined MEAET homology model.

The plot shows the distribution of backbone dihedral angles for all non-glycine residues. The percentage of residues located in the most favored regions was 51.6%, indicating a good model quality.

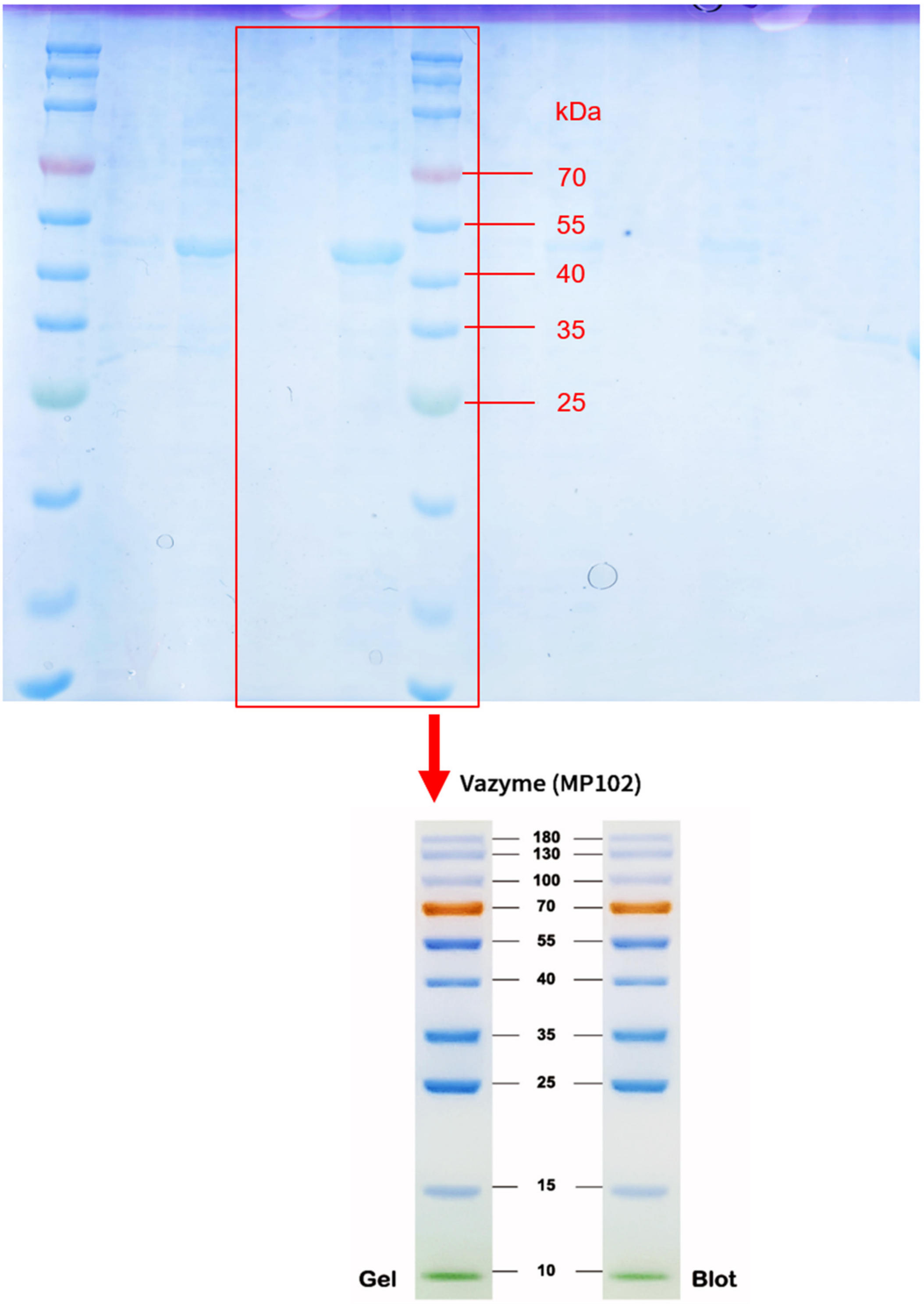

Supplementary Figure S2. Uncropped SDS-PAGE gel of the purified recombinant proteins.
The full-length original gel corresponds to the cropped region shown in Figure 2A, with 10 μL sample loading per well.

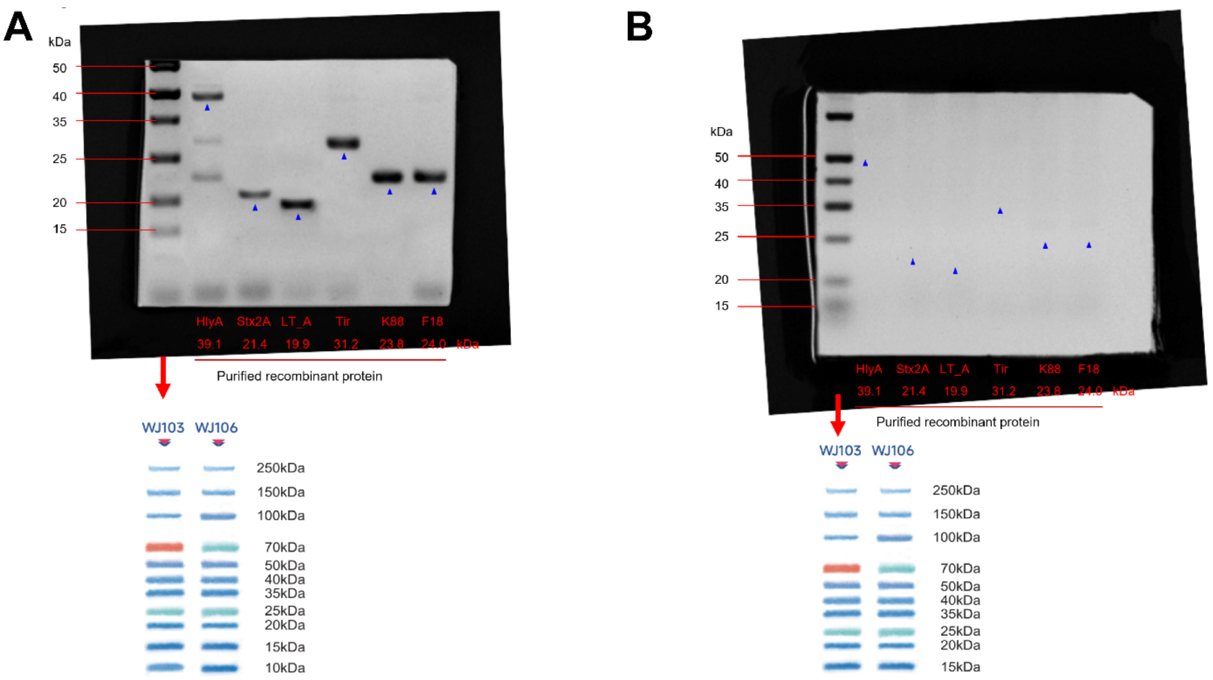

Supplementary Figure S3. Uncropped Western blot images of the six recombinant proteins. (A) Blots probed with hyperimmune serum raised against MEAET. Each recombinant protein (HlyA, Stx2e, LT, Tir, K88/F4, and F18) was detected with the positive serum. (B) Blots probed with negative control serum. No specific bands were observed. The full-length original blots correspond to the cropped regions shown in Figure 2B. The blots were developed with ECL substrate and exposed for 3 seconds. Molecular weight markers are indicated on the left. Red boxes indicate the cropped regions presented in the main figure.
